# Pangenome Analysis of *Burkholderia pseudomallei*: Genome Evolution Preserves Gene Order despite High Recombination Rates

**DOI:** 10.1371/journal.pone.0140274

**Published:** 2015-10-20

**Authors:** Senanu M. Spring-Pearson, Joshua K. Stone, Adina Doyle, Christopher J. Allender, Richard T. Okinaka, Mark Mayo, Stacey M. Broomall, Jessica M. Hill, Mark A. Karavis, Kyle S. Hubbard, Joseph M. Insalaco, Lauren A. McNew, C. Nicole Rosenzweig, Henry S. Gibbons, Bart J. Currie, David M. Wagner, Paul Keim, Apichai Tuanyok

**Affiliations:** 1 Department of Biological Sciences, Northern Arizona University, Flagstaff, AZ 86011, United States of America; 2 Menzies School of Health Research and Infectious Disease Department, Royal Darwin Hospital. Darwin, Northern Territory, Australia; 3 BioSciences Division, Edgewood Chemical Biological Center, Aberdeen Proving Ground, MD, United States of America; 4 Department of Infectious Diseases and Pathology, University of Florida, Gainesville, FL, United States of America; Columbia University, UNITED STATES

## Abstract

The pangenomic diversity in *Burkholderia pseudomallei* is high, with approximately 5.8% of the genome consisting of genomic islands. Genomic islands are known hotspots for recombination driven primarily by site-specific recombination associated with tRNAs. However, recombination rates in other portions of the genome are also high, a feature we expected to disrupt gene order. We analyzed the pangenome of 37 isolates of *B*. *pseudomallei* and demonstrate that the pangenome is ‘open’, with approximately 136 new genes identified with each new genome sequenced, and that the global core genome consists of 4568±16 homologs. Genes associated with metabolism were statistically overrepresented in the core genome, and genes associated with mobile elements, disease, and motility were primarily associated with accessory portions of the pangenome. The frequency distribution of genes present in between 1 and 37 of the genomes analyzed matches well with a model of genome evolution in which 96% of the genome has very low recombination rates but 4% of the genome recombines readily. Using homologous genes among pairs of genomes, we found that gene order was highly conserved among strains, despite the high recombination rates previously observed. High rates of gene transfer and recombination are incompatible with retaining gene order unless these processes are either highly localized to specific sites within the genome, or are characterized by symmetrical gene gain and loss. Our results demonstrate that both processes occur: localized recombination introduces many new genes at relatively few sites, and recombination throughout the genome generates the novel multi-locus sequence types previously observed while preserving gene order.

## Background

*Burkholderia pseudomallei* is a Gram-negative, soil-dwelling bacterium that is the causative agent of melioidosis, a disease endemic to Southeast Asia and northern Australia. Ecologically, *B*. *pseudomallei* inhabits primarily soil and water [1, 2] with most infections occurring in wet agricultural areas [2]. The ability of *B*. *pseudomallei* strains to infect by the aerosol route, the broad environmental and host range, and the general hardiness of the organism have caused such strains to be listed as a Tier 1 Select Agent due to potential bioterrorism risks [3] The host range is very broad including birds, crocodiles, and marsupials, but the predominant hosts are placental mammals [4]. The genome is large, consisting of two chromosomes of 4.07 and 3.17 Mbp, respectively [5]. Genomic islands (GIs) are gene coding regions that are unique in one of a very few strains and identified by comparing genomes or by their differential GC/AT ratio. They many contain a few or many genes and are thought to be a result of horizontal gene transfer from other bacteria, often ones that are only somewhat related. They are key features of the *B*. *pseudomallei* genome, accounting for an average of approximately 5.8% of individual genomes and are a major source of genomic diversity among different strains [6] with the presence of particular genes being associated with pathogenicity in humans [7]. GIs have also been shown to contain genes associated with metabolism, and, most commonly, genomic mobility with 80% of GIs containing at least one transposase [6]. Indeed, GIs themselves serve as ‘hotspots’ for insertion of foreign DNA [6].

Although GIs may account for a relatively large amount of observed genetic diversity especially relative to their size, acquisition of foreign DNA is not restricted to those sites. *B*. *pseudomallei* genome is widely considered to be ‘open’ [6] (sequencing new strains is always expected to lead to the discovery of new genes), with very high levels of lateral gene transfer [8] generating the large and moderately diverse genome observed. In contrast, bacteria that have ‘closed’ genomes, like *Mycobacterium tuberculosis*, are not the recipients of horizontal gene transfers. Using comparative genomic hybridization (CGH) microarrays, Sim et al. [7] found that 750 out of 5369 genes (14%) in the *B*. *pseudomallei* K96243 strain were not present in each of 94 other strains and considered these to be accessory genes. They therefore considered the remaining 86% to be the core genome, the portion of the genome that includes genes responsible for basic aspects of the biology of the species [9]. Their study, however, only used strains from a relatively narrow geographic range, possibly missing some of the genomic variability that may exist across the entire range of the species. Nevertheless, they found that almost one third of the 750 accessory genes were localized to genomic islands within the reference K96243 genome [7]. However, this means that over 500 genes are considered accessory and are not located within a known GI. Moreover Pearson et al. [8] demonstrated high levels of diversity in *B*. *pseudomallei* in datasets from MLST (Multi-Locus Sequence Typing) analysis suggesting that genetic exchange has not been limited to GIs. These conserved MLST sites are located at scattered locations along the genome and suggest that homologous exchange occurs extensively throughout the genome and includes presumably core ‘housekeeping’ genes.

Haegeman and Weitz [10] proposed models of genome evolution in which the frequency with which a gene appears in a collection of genomes informs the models. Specifically, they found that if genomes could acquire genes from the environment and those genes were inserted randomly into the genome (neutral model A of Haegeman and Weitz, [10]), then the expected frequency distribution would be U-shaped, with many genes being strain-specific, few genes being common but not universal, and many genes having orthologs in all strains. In such a model, there is no ‘core’ genome, as any gene may be exchanged with the environmental pool. Additionally, the large numbers of genes with orthologs in all strains is due to either low gene transfer or by sampling only a few strains and is not necessarily indicative of any of these genes being essential for the organism. However, non-neutral models in which some portions of the genome were relatively resistant to accepting new genetic material and a very small portion in which genes were readily incorporated, generally fit the data better for 6 species studied by Haegeman and Weitz [10]. These types of models incorporate a core genome that is strictly prevented from exchanging genes with the environment (model C)[10] or a core genome in which recombination is possible, though unlikely (model D).

Koonin et al. [11] classified horizontal gene transfer events into 3 categories: i) acquisition of a new gene that is not homologous to other genes in the genome or pangenome, ii) acquisition of a paralog with no or only distant evolutionary history, and iii) homologous recombination in which acquisition of an ortholog is followed by displacement of the ancestral gene (reciprocal displacement). The first two cases involve disrupting the gene order of the recipient genome with respect to its ancestral state whereas the last usually does not unless other genes are transferred as well and the transfer is not completely reciprocal [12]. For the most part, however, recombination rates are positively related to sequence similarity [13–15] and homologous recombination among closely related strains retains the original gene order.

The *B*. *pseudomallei* genome, therefore, appears to have somewhat paradoxical organization. On the one hand, there appears to be extensive conservation of gene order when genomes are compared [16], suggesting that horizontal gene transfer rates are low or at least occur in such a way that gene order is preserved. This may happen even with extensive homologous recombination or by integrating the foreign DNA into regions in which the ancestral gene order has already been disrupted. On the other hand, high levels of lateral gene transfer [8] that is not strictly localizable to few sites [7, 8] has been observed, which would be expected to dramatically reduce the conservation of gene order unless the recombination is almost exclusively homologous.

There are two main goals of this paper. The first is to describe the pangenome of *B*. *pseudomallei* using a geographically broad selection of 37 genomes. We demonstrate that, as has been suspected, the genome is open and that the core genome size, expected to decrease as the number of sequenced strains increases, is 4568±16 genes. Secondly, we investigate the role of horizontal gene transfer in breaking up gene order and find that gene transfer of dissimilar DNA is likely to occur at only a few sites, presumably GI locations, and that this allows gene order to be preserved widely across the genome.

## Methods

### Classification into homology groups and description of pangenome

Genome sequences were obtained either from GenBank or by using standard 454 pyrosequencing methodology and chemistry [17]. For the pyrosequencing, genomic DNA was shotgun-sequenced to a minimum of 25x average sequence coverage using the Roche 454 GS-FLX Titanium sequencers according to manufacturer's instructions. Each draft shotgun assembly was generated *de novo* using Newbler GSAssembler v2.3.

For this study, we analyzed 37 *B*. *pseudomallei* genomes, 10 of which were finished, 3 of which are drafted into 2 chromosomes (hereafter referred to as ‘drafted’), and 24 of which were unfinished (Table 1; See also S1 File for Genbank accession numbers). For each, we predicted genes using the software Prodigal [18] and translated DNA to amino acid sequences using myRAST, a downloadable software package that uses the RAST servers [19]. This also provided a predicted function of each gene, where possible. Although some of the genomes included in our analysis have been previously described and annotated, our analyses required internally consistent gene-calling and annotation: therefore myRAST was utilized to annotate all genomes used in this work. We filtered the resulting predicted proteins to include only those with more than 80 amino acid residues or those to which a function could be assigned by myRAST. This threshold was chosen as shorter ORFs are less likely to be protein encoding genes (only approximately 1.3% of predicted proteins in the NCBI protein database are shorter than 80 residues), are unlikely to provide positive matches against sequence databases, and may artificially inflate the size of the pangenome.

**Table 1:** Number of genes identified by myRAST.

					Homologous Groups (HGs)			
Isolate	Origin^1^	Contigs	Total genes	Retained genes^2^	Total	Extended core	Character	Strain-specific
1026b^3^	Thailand/C	2	5961	5755	5662	4731	891	40
1106a^3^	Thailand/C	2	5759	5580	5510	4736	756	18
1258a	Thailand/C	340	5723	5520	5499	4550	829	120
1710b^3^	Thailand/C	2	5979	5760	5691	4734	901	56
354e	Thailand/C	338	6039	5798	5762	4722	883	157
406e	Thailand/C	2	5997	5796	5511	4649	790	72
4900CFPatient1	Brazil/C	487	6020	5791	5767	4704	852	211
576	Thailand/C	21	5959	5752	5676	4724	841	111
Bp22^3^	Singapore/C	2	6003	5676	5686	4521	708	457
BPC006^3^	China/C	2	5874	5742	5623	4730	825	68
Gu1909a	Thailand/C	413	6107	5882	5865	4649	886	330
K96243^3^	Thailand/C	2	5948	5731	5651	4735	868	48
MSHR1655^3^	Australia/C	2	5823	5633	5480	4553	840	87
MSHR1950	PNG/C	323	6259	6002	5960	4641	779	540
MSHR305^3^	Australia/C	2	6140	5938	5850	4720	946	184
MSHR346^3^	Australia/C	2	6048	5847	5792	4718	954	120
MSHR465a	Australia/C	229	5859	5680	5660	4723	844	93
MSHR668^3^	Australia/C	2	5795	5613	5563	4679	736	148
NAU14B6	Australia/S	239	5979	5779	5755	4724	977	54
NAU20B16	Australia/S	543	6300	6063	6039	4656	1033	350
NAU44A6	Australia/S	243	6082	5875	5848	4714	994	140
NCTC13178	Australia/C	459	6365	6127	6102	4664	1091	347
NCTC13179	Australia/C	468	6264	6043	6014	4691	1076	247
NCTC13392	Thailand/C	48	5893	5682	5634	4730	832	72
PB08298010	USA/C	354	6129	5912	5886	4716	907	263
PHLS9	Pakistan/C	192	5947	5734	5709	4729	955	25
Pakistan9	Pakistan/C	71	5976	5766	5707	4681	910	116
Pasteur52237	Vietnam/C	2	6088	5856	5656	4685	830	141
RF43BP22	Thailand/S	121	5911	5718	5692	4733	941	18
RF67BP1	Thailand/S	32 9	5963	5748	5723	4698	871	154
RF6BP15	Thailand/S	262	5989	5766	5744	4714	916	114
NRF80Bp1	Thailand/S	254	5680	5491	5467	4503	791	173
RF85Bp37	Thailand/S	320	5977	5770	5750	4685	891	174
RNS3Bp1	Thailand/S	229	6024	5801	5772	4720	961	91
RNS7Bp6	Thailand/S	281	5976	5758	5735	4701	889	145
S13	Singapore/S	2	6081	5873	5647	4714	838	95
Songkhla34W2	Thailand/W	269	6036	5806	5784	4729	976	79
Mean			5999	5786	5726	4687	887	152

^1^ Country/source where C = clinical, S = soil, W = Water.

^2^ Genes retained were either >80 amino acids or were annotated by myRAST with functions other than “unidentified orf” or “hypothetical protein”.

^3^ Finished genomes.

We located homologous genes from the 37 isolates using a slightly modified version of OrthoMCL [20]. This algorithm uses an all-vs-all BLASTp search to calculate similarity among the predicted proteins and identifies groups of homologs and paralogs. Our modifications forced OrthoMCL to only consider matches that were at least 80% similar and in which the shorter amino acid sequence was at least 80% the length of the longer sequence. Because we used a non-templated *de novo* assembly of 19 of the genomes and such assemblies often fail to distinguish paralogs, and because the output of OrthoMCL groups orthologs and paralogs together, we use the terms ‘genes’ to refer to genes predicted by myRAST (which may combine the sequences of paralogs) and ‘homologous groups’ (HGs) to describe groups of genes (orthologs and paralogs) with similar amino acid sequences.

RAST annotations and their functional categories were re-linked to the sequences. Many genes were classified by RAST as having more than one role in which case genes were counted once for each functional role they play in the cell. We used a *χ* ^2^ test to test the null hypothesis that the genes in each of 27 functional groups (excluding genes with no functional group assigned) were distributed evenly among the core (present in at least 35 isolates; see also below for discussion of this definition), character (present in between 2 and 34 isolates, inclusively), and unique (present in only a single isolate) classifications. Thus, the test compared the observed numbers of each type (core, character, unique) of gene in each functional group in a 27x3 contingency table. Residuals from this analysis indicated whether particular functional groups were over (positive residuals) or underrepresented (negative residuals) by core, character, or unique genes.

In some cases, members of a single homology group (as determined by OrthoMCL) were annotated differently by myRAST. When this was the case, we assigned the group the function of the majority of group members except when that majority was labeled by myRAST as a “hypothetical protein”. In such cases the “hypothetical protein” label was retained only if more than 75% of the group members were so labeled; otherwise the next most common functional annotation was used.

### Size of core and pangenome

To predict the size of the core and accessory genome, we conducted a rarefaction analysis as described by [21]. The order of strains in which genes are discovered was permuted and for each permutation, genes were identified as core if for *n* genomes, the gene was present in at least *n-2* strains (see also above). We also conducted the analysis using the stricter definition of “core” in which genes must be present in all strains to be considered. As described by Tettelin et al. [21], the number of core genes identified is a decaying function, which we fit to the data using non-linear regression (see Eq 1 below).

The rate of the decline in the number of new genes discovered as more genomes are sequenced can be used to determine whether the genome is ‘open’ or ‘closed’ [22]. If the number of newly discovered genes for the *n*-th genome is *N*(*n*) = *An* ^*ξ*^ then the pangenome is open for *ξ*>−1 and converges to a constant for *ξ*<−1 [22]. We used linear regression to fit our data to this function.

### Genome evolution

In order to calculate the parameters to fit the models described by Haegeman and Weitz [10], we used the MATLAB scripts provided in their paper. We used the empirical distribution of homologous groups present in 1 to 37 genomes. We calculated genome fluidity as described by Kislyuk et al. [23], using their jackknife method to obtain the variance of the estimate.

Methods of calculating synteny (*σ*) that simply consider whether genes are located on the same chromosome in a pair of genomes (e.g. [24]) are not appropriate for calculating gene order conservation in bacteria, which typically have only 1 or a few chromosomes. In order to accommodate both finished/drafted genomes with only 2 chromosomes and unfinished genomes with potentially several hundred contigs, we used a sliding window similar to that used by Huynen and Bork [25] to analyze adjacency of genes. For each homolog *a* in genome *A*, we found the homolog *b* in genome *B* and calculated the proportion of homologs, within a given window size, from *a* that are also present in the same window around *b*. With a window size of 3 (one gene on each side of the target gene), *σ* is simply the probability that an adjacent gene in genome A is also adjacent in genome B. Because paralogs may be part of homologous groups, we searched all possible paralogs of *b* in B and selected the one with the most common genes within the window. By using a small window of size 3, we minimized the negative effect on gene order conservation that having the genome broken into many contigs creates. By randomizing the gene order of genome *A*, we confirmed the expectation that *σ* ranges from approximately 1/*g* to 1 where *g* is the number of homologous groups in the genome.

## Results and Discussion

There are 221,953 genes predicted by myRAST for the 37 genomes studied (Table 1). Of these, 7,889 were discarded as they have fewer than 80 amino acids and were classified by myRAST as hypothetical proteins or unidentified ORFs. Although there were fewer short (<80 amino acids) proteins and ORFs identified in the genomes that were finished (or drafted to 2 contigs) than in those with more than 2 contigs ($\bar{x}\;=\;292$ vs 332; Welch’s *t* = 3.8827, *df* = 31.27, *p*<0.001), there was no difference between the number of retained genes in the finished (or assembled to 2 contigs) genomes compared to those with more contigs ($\bar{x}\;=\;5804$ vs 5755 respectively; Welch’s *t* = 1.168, *df* = 34.5, *p* = 0.252). These data suggest that the breaks between contigs generate an excess of short open reading frames (ORFs) but this does not significantly reduce the number of longer, identifiable genes. The number of large and/or identifiable genes in strain K96243 (5,731) is within 5% of the 6,010 genes found in a re-annotation of that genome by Nandi et al. [16].

### Core and pangenome: content and size

Genes comprising the extended core genome (*sensu* Lapierre and Gogarten [26]) were identified by their presence in at least 35 of the 37 strains. We used this lower threshold rather than all 37 (hereafter the ‘strict core genome’) because two strains, NRF80Bp1 and MSHR1655, which were isolated from the environment and a human patient, respectively, have each experienced significant gene loss [27]. In the case of NRF80Bp1, this gene loss includes the *bimA* and Type VI secretion system cluster I virulence locus and a pathogenicity island including cluster III of the Type III secretion system [28, 29]. In both MSHR1655 and NRF80Bp1, the loss of these key virulence genes has also likely decreased or eliminated their ability to infect a new human host.

**Fig 1:**
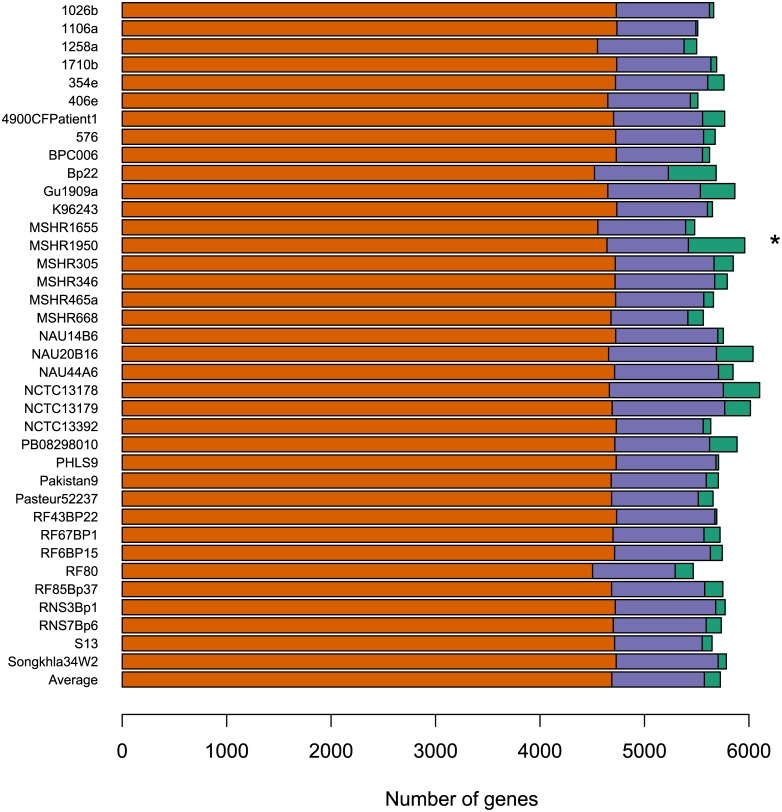
Genomic composition. Number of core (orange bars), character (purple bars), and unique (green bars) genes in 37 genomes. * MSHR1950 contains a 131kb plasmid.

Of the 213,562 genes at least 80 amino acids in length or to which myRAST could ascribe a function, OrthoMCL constructed 13,799 homologous groups (S2 File). On average, the strains have 5,726 predicted HGs, 4,687 of which are part of the extended core, 152 are strain-specific, and the remaining 887 are character HGs (present in 2 to 34 genomes, inclusively; Table 1, Fig 1). Thus, approximately 82% of the genes were classified as extended core genes in the average genome, a figure lower than the 86% estimated by Sim et al. [7]. There is large variation in the number of strain-specific genes, ranging from 18 (strains 1106a and RF43BP22) to 540 (strain MSHR1950). The latter genome is also characterized by the presence of a 131kb plasmid, which helps account for this large number of strain-specific genes.

**Fig 2:**
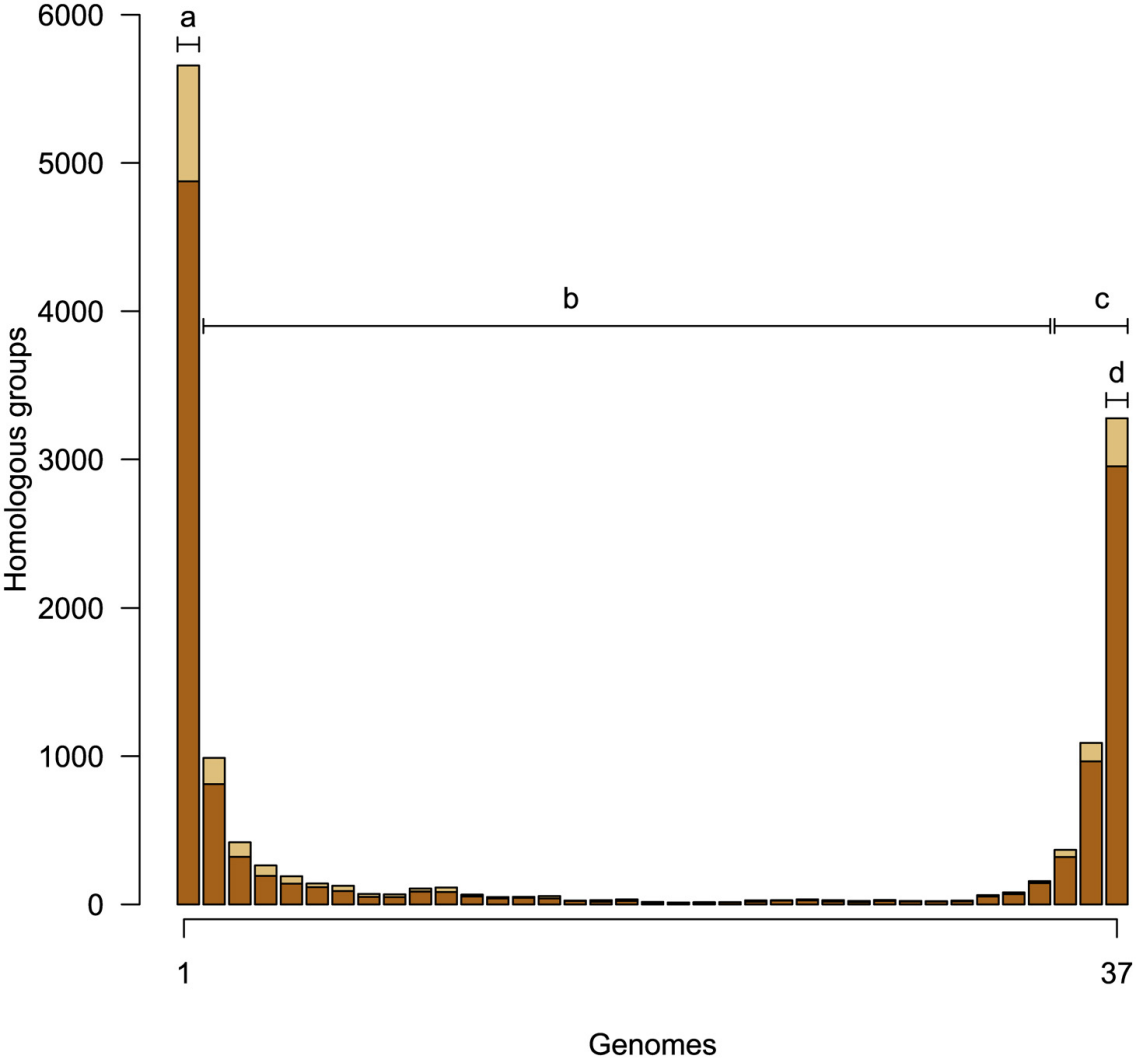
Frequency distribution of homology groups. Dark shading indicates HGs for which myRAST identified a function, lighter shading were identified only as “hypothetical proteins”. Strain-specific genes are indicated by the leftmost bar (a), and character HGs (b) are between strain-specific genes and HGs of the extended core (c). The strict core genome is made up of HGs present in all 37 strains (d). Although relatively few genes per genome ($\bar{x}\;=\;153$) are strain-specific, the cumulative total across the pangenome is high.

**Fig 3:**
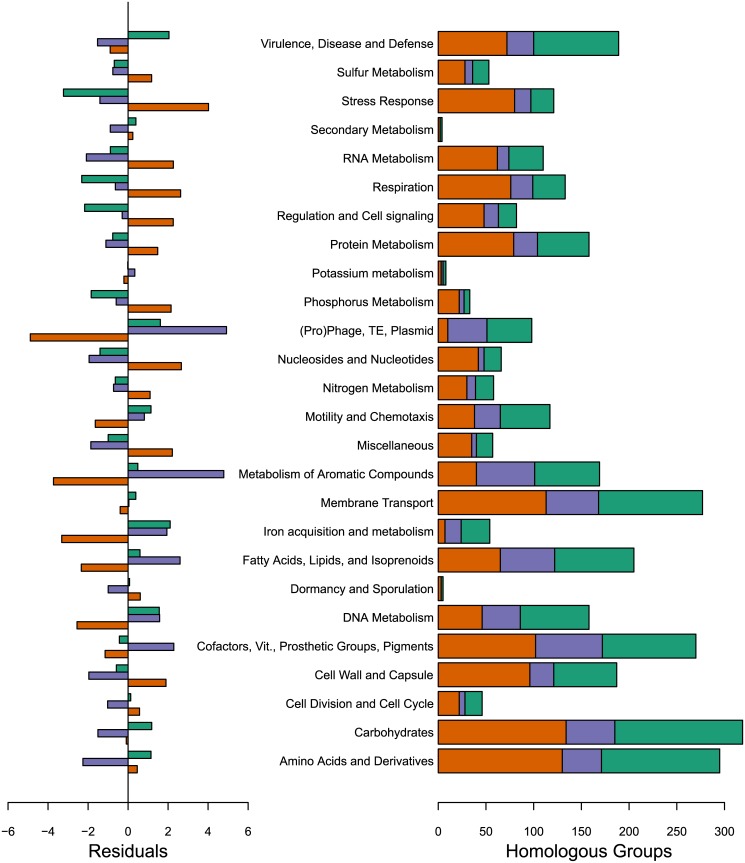
Distribution of gene functions. Number of core (orange), character (purple), and strain-specific (green) genes in each of the RAST functional groups (right). Proteins labeled “hypothetical”, “putative”, or without a functional category were not included. *Χ* ^2^ residuals are shown on the left (see text for details).

As with other species, particularly naturally competent ones, the frequency distribution of homologous groups is U-shaped [10, 30] with most groups being strain-specific or part of the extended core (Fig 2). The distribution of functional categories as annotated by myRAST is shown in S2 File. Chi-square analysis showed that the core, character, and unique HGs were not distributed at random among the RAST categories, where such categories could be identified (*χ* ^2^ = 272, *df* = 50, *p*<<0.001). *χ* ^2^ residuals suggest that this is driven primarily by an overrepresentation of some metabolic functions among the core and, notably, an underrepresentation among the core HGs of homologs associated with other metabolic functions, mobile elements, disease, and motility (Fig 3). This is not surprising as the numerous genomic islands present in *B*. *pseudomallei* are comprised largely of genes associated with mobile elements and pathogenicity [6]. Additionally, mutually exclusive gene clusters *B*. *thailandensis*-like flagella and chemotaxis gene cluster (BTFC) and *Yersinia*-like fimbrial gene cluster (YLF) are composed of many copies of motility and fimbriae genes, respectively [31], resulting in an overrepresentation of these categories among the character genes.

**Fig 4:**
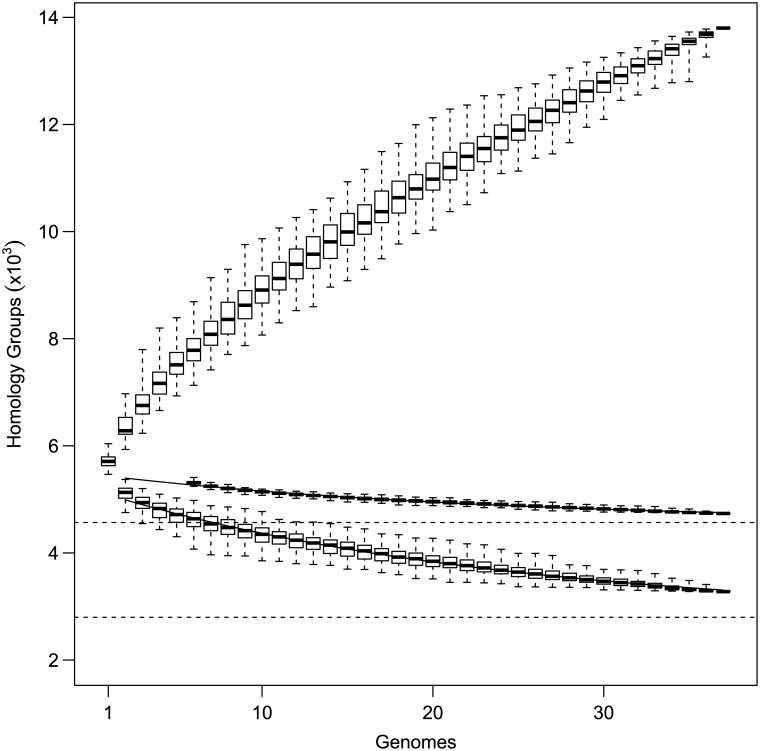
Pan and core genome size. The discovery order of genomes was permuted 100 times with the total number of genes discovered shown in upper series, and number of core genes below. The middle series considers genes to be core if they are present in at least *n*−2 of the genomes (extended core). The bottom series considers them to be core only if present in every strain (strict core). Fitted lines for core homology groups are *F*_*c*_(*n*) = *κ*_*c*_ exp[−*n*/*τ*_*c*_]+Ω where Ω is the estimated core genome size (dotted lines: 4,568 homology groups for the extended core, and 2,798 for the strict core). Boxes show medians, interquartile range, and full range of data.

After sequencing 37 genomes, the extended core consisted of 4,736 HGs and the strict core genome consisted of 3,278 HGs (Fig 4; note that the number of extended core HGs is slightly larger than the average number of HGs, 4,687, given above as not all HGs are present in each genome; see methods). In order to estimate the core genome size expected as more genomes are sequenced, we simulated 100 permutations of the sequencing order for the 37 genomes used here [21]. As described by Tettelin et al. [21], the number of core genes identified after sequencing *n* genomes is

$$F_{c}(n)=\kappa_{c}\text{exp}[-n/\tau_{c}]+\Omega$$ (1)

Where *κ*_*c*_ gives the magnitude of the decay and *τ*_*c*_ is the rate at which the curve approaches the asymptotic core genome size Ω. We fit Eq 1 to our permuted data and found that the extended core genome size was Ω = 4568±16 HGs as *n*→∞ and the strict core genome size was Ω = 2798±59 HGs (Fig 4). The small difference between the number of extended core HGs found and the number expected as more genomes are sequenced suggests that 37 genomes used here is likely to provide a good estimate of the global extended core genome.

As more genomes are sequenced, the number of new genes discovered (*N*(*n*)) decreases with each new genome (*n*) according to the equation

$$N(n)=An^{\xi }$$ (2)

**Fig 5:**
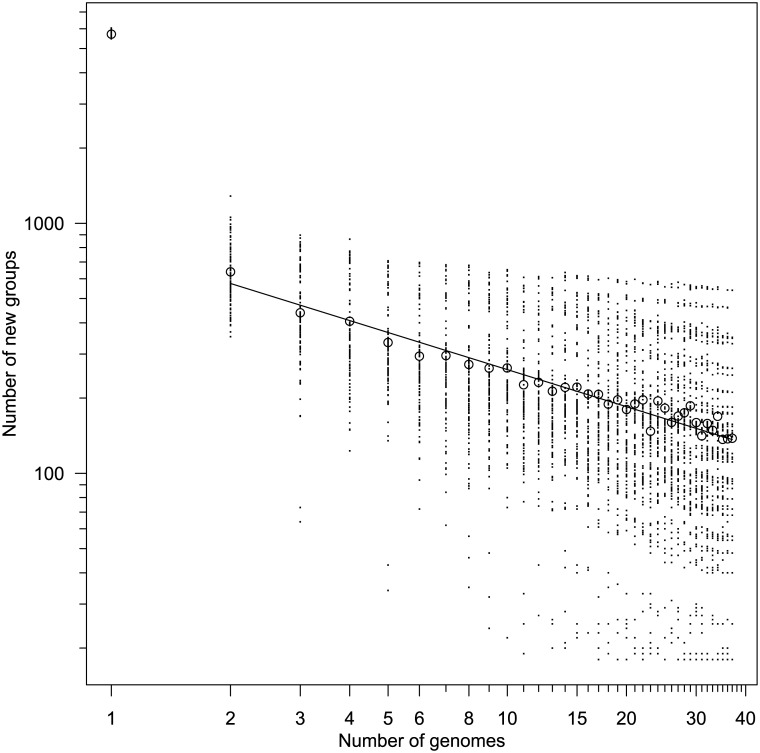
Number of new genes added to pangenome with addition of each genome. The order genomes were added was permuted 100 times, with the mean shown as circles. The fitted line is *N*(*n*) = 809*n* ^−0.49^ and excludes the data from the first genome.

With *ξ* indicating an open pangenome if *ξ*>−1 and a closed pangenome that converges to a constant if *ξ*<−1 [22]. Using the permutations described above, we found that *N*(*n*) = 809*n* ^−0.49^, confirming that the pangenome is indeed open and sequencing new strains is always expected to yield new genes (Fig 5). On average, the addition of the 37^th^ genome added 136 genes to the pangenome. This can also be seen in the nearly linear increase in pangenome size in Fig 4.

Estimating the pan and core genome sizes using the rarefaction analysis above relies on the ability to detect rare genes in the case of estimating the pangenome, and the ability to detect genomes with unusual genotypes in the case of estimating the size of the core genome [23]. Genome fluidity (*ϕ*) has been proposed as a robust measure of genomic dissimilarity that does not require large numbers of genomes to be sequenced as it does not rely on finding such rare genes or genomes [23]. It is essentially the average of the ratio of the number of genes that are unique in each of a pair of genomes to the number of genes that have homologs [23]. Haegeman and Weitz [10] argued additionally that genome fluidity is a proxy for the relative importance of gene uptake in genomic composition. We calculated *ϕ* = 0.107 with a standard deviation of 0.006. This estimate is lower than that calculated for any other bacterium by Kislyuk et al. [23] with the exception of *Bacillus anthracis*, although direct comparison between studies is difficult (see below).

Kislyuk et al. [23] showed that relative values of genome fluidity are robust to changes in parameter values (used in BLAST searches to construct homology groups). True as this may be, it fails to provide a metric that can be compared among studies because even though the relative ordering of species may be robust, the value itself is highly dependent on the parameters used, requiring identical methodology for direct comparison. Indeed, their own analysis (Fig 5 in [23]) shows the large range of estimated fluidity values with higher values resulting from more stringent BLAST search parameters. Such discrepancy underscores both the need for a metric of (dis)similarity that is insensitive to parameter values and a recognition that comparison of values across studies needs to be done with caution.

### Frequency distributions of genes

**Fig 6:**
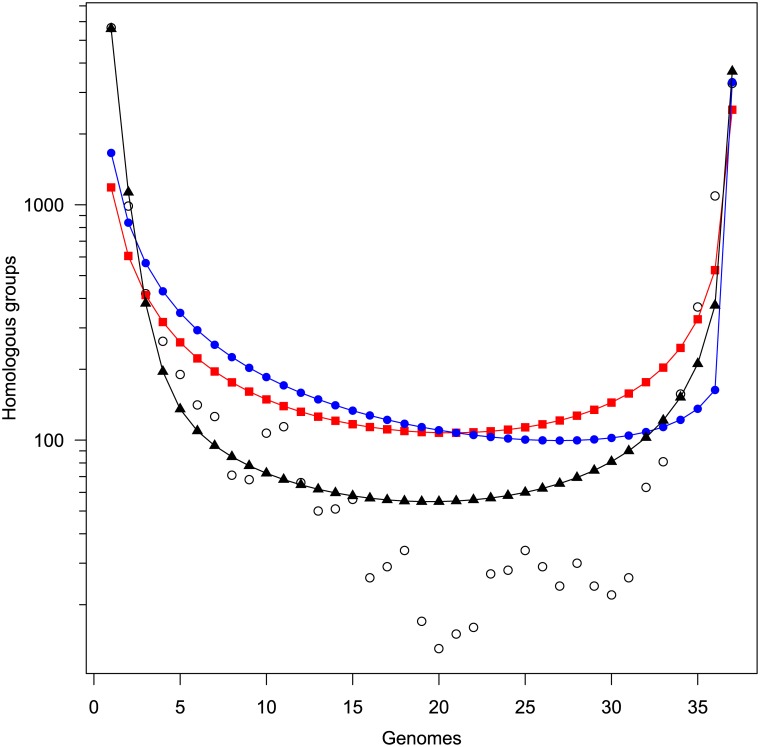
Distribution of genes and fit of models described by Haegeman and Weitz [10]. Open circles are data from this study, with fitted lines according to models A (red squares), C (blue filled circles), and D (black triangles). See Table 2 and text for descriptions of models and parameters.

Haegeman and Weitz [10] developed several null models of genome evolution. We used MATLAB scripts provided by the authors to estimate parameters of the models. We found that the simplest (neutral) model (model A), in which any portion of the genome may pick up DNA from the environment, provided a relatively poor fit to the data with the gene transfer parameter *θ* = 0.2025 (Table 2 and Fig 6). The gene transfer parameter is difficult to interpret as it depends on the population size, reproductive rate, and gene transfer rate, which are essentially unknown, and multiple combinations can provide the same estimate of *θ* [10]. We therefore evaluated the models relative to one another. In the non-neutral model C, a fraction (*λ*_1_) of the genome is considered the rigid core and does not exchange genes with the environment. The rest of the genome exchanges genes the same as in model A. As with model A, the fit was relatively poor and suggested that only 54% of the genome constituted the core with the rest exchanging DNA at about thrice the rate as in model A. Model D, which allows for a low rate of recombination to occur in most of the genome, provided the best fit with our data, with 96% of the genome being a relatively rigid core with a low rate of exchange with the environment, and the remaining 4% of the genome exchanging DNA very freely with the environment.

**Table 2:** Model fit parameters. Models described by Haegeman and Weitz [10]. Model A: neutral model with all genes exchanged with environment with parameter *θ*_1_. Model C: Genome has a fraction (*λ*_1_) of the genome that is rigid (the core), and the rest exchanges genes with the environment with parameter *θ*_1_. Model D: Similar to model C except the core exchanges genes with the environment with parameter *θ*_2_. The distance from the model fit to the data for *B*. *pseudomallei* is given by Δ, with smaller numbers signifying better fit.

	*θ*_1_	*λ*_1_	*θ*_2_	Δ
Model A (no core)	0.2025			71.18
Model C (rigid core)	0.6174	0.5351		65.90
Model D (flexible core)	72.4334	0.9644	0.0986	11.19

Genomic islands are known to be recombination hotspots sometimes with several recombination events overlapping one another [6]. The 5 strains considered by Tuanyok et al. [6] have an average total length of 417.93 kbp of genomic islands and an average genome size of 7.23 Mbp amounting to 5.8% of the genomes within genomic islands. This result is very similar to the 4% predicted to have high fluidity by model D, especially considering the model also allows for some low level of recombination in the rest of the genome.

### Conservation of gene order

The large extent to which the *B*. *pseudomallei* genome is open and its readiness in taking up DNA from the environment suggests that gene order is likely to be disrupted by recombination events. However, gene order across the genome may be preserved if recombination is completely reciprocal or occurs almost exclusively at only a small number of sites (such as GIs) since gene order at most other sites would not be disrupted, even after extensive recombination.

**Fig 7:**
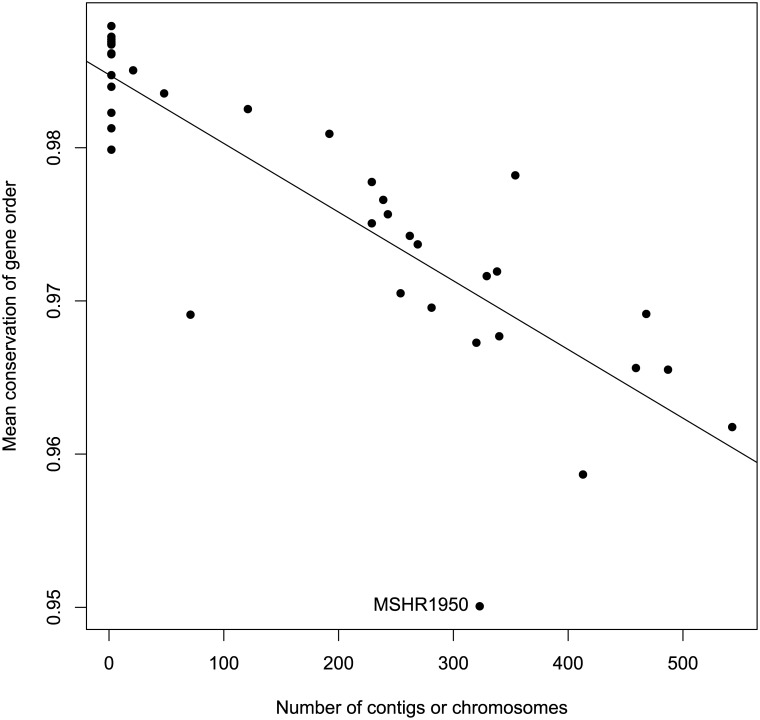
Conservation of gene order among pairs of genomes. Average conservation of gene order for each genome is negatively related to the number of contigs.

**Fig 8:**
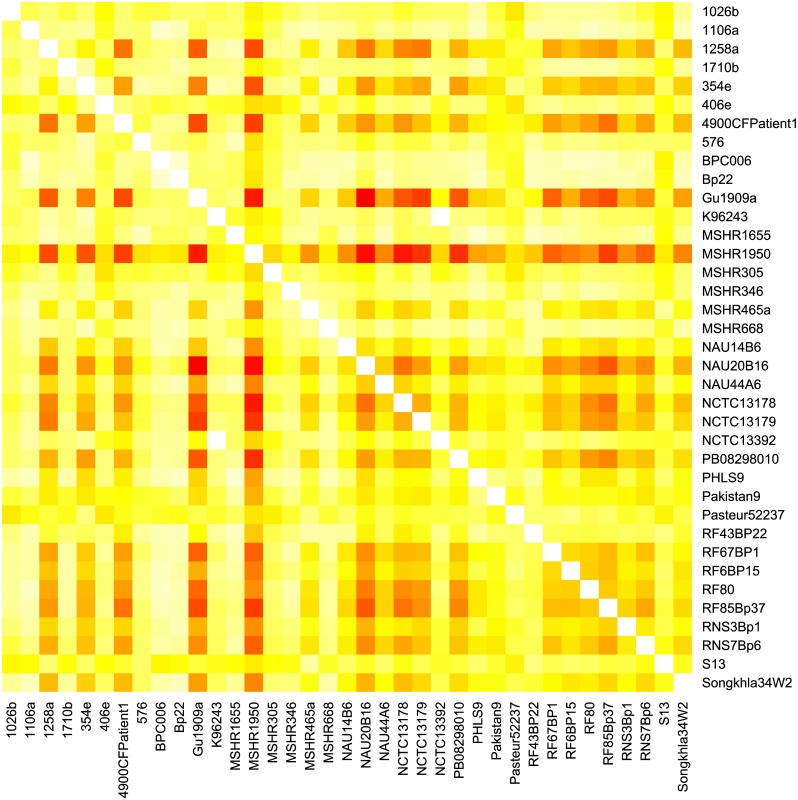
Pairwise values for gene order conservation. Reds indicate relatively low similarity of gene order and yellows, higher. All values are in the range 0.9089<*x*<0.9980.

Because many of the genomes in our study were unfinished, we used a method of measuring gene order conservation similar to that used by Lovell et al. [32] and Huynen and Bork [25] that is only very weakly dependent on the number of contigs (or chromosomes) in the assembled genomes (Fig 7). We calculated *σ*, the probability in pairs of genomes that homologs adjacent in one strain are also adjacent in the other. Values of *σ* range from 1/*g* when the gene order of *g* genes is completely randomized, to 1 when the genes from the two strains are in the same order. The mean value of *σ* in this study was 0.9765 (range: 0.9089–0.9980; Fig 8) indicating that the gene order of adjacent genes is disrupted in only 2.4% of orthologous gene pairs. Though tentative due to the fragmentary nature of most genomes studied, this confirms previous qualitative results [16] showing large blocks of genes in the same order across genomes.

**Fig 9:**
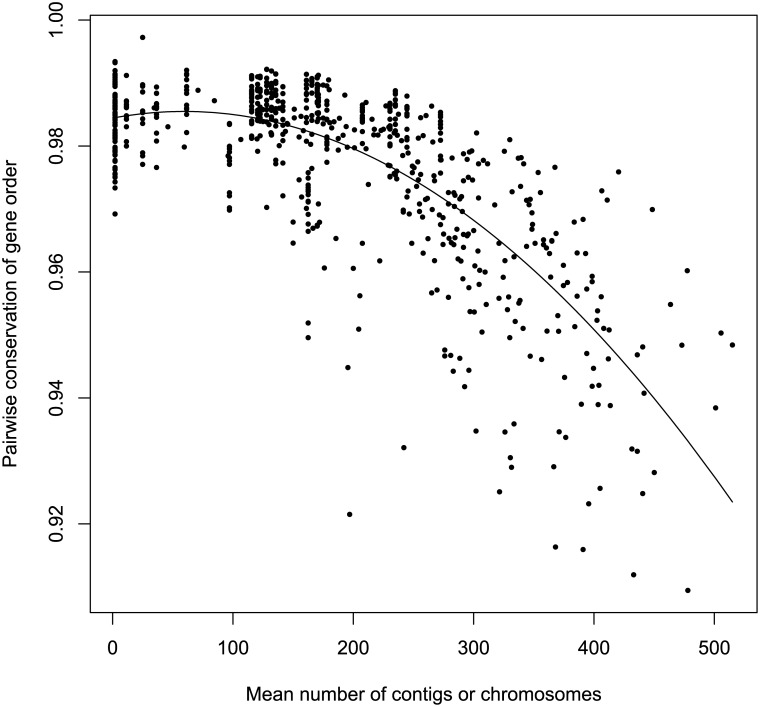
Influence of number of contigs on gene order conservation. Number of contigs is the mean number of contigs of the pair of isolates. Fitted line is *y* = 0.9844+3.5×10^−5^ *x*−3.0×10^−7^ *x* ^2^.

Because we used unfinished genomes in this study, there is a possibility that the measure of gene order conservation that we used is related to the number of contigs in the assembly. Indeed, linear regression (Fig 7) of the average gene order conservation of a genome on the number of contigs shows that there is a negative relationship (*p*<0.001, slope = −4×10^−5^) and it accounts for a large portion of the observed variance (*R* ^2^ = 0.704). MSHR1950 has markedly lower average similarity in gene order (Fig 8). This may largely be explained by the presence of a plasmid and its fairly basal position in the species tree, resulting in relatively large evolutionary distances separating it from other isolates (T. Pearson, pers. comm.). Comparison of pairwise gene order conservation values (rather than average values over all pairs) to mean number of contigs shows that the number of contigs has little influence on conservation of gene order for pairs of isolates with an average of 200 contigs or fewer (Fig 9).

### Forces that drive the evolution of *Burkholderia pseudomallei*

The pan-genome analysis combined with previous data sets suggests that the phenotypic diversity in *B*. *pseudomallei* is greatly influenced by the influx (or removal) of a plethora of accessory genes via the mobility of genomic islands. Insertion of “foreign” DNA into a small number of hotspots helps explain the large diversity of strain-specific and character genes as well as the large pan-genome size. The transfer of such unrelated DNA is often marked by differing GC content and dinucleotide bias [12–14, 33–35] and is characteristic of genomic islands in this species [5, 6].

Conversely, the phylogenetic diversity appears to be affected by a homologous recombination system that results in scrambling of the core genome; e.g., the MLST sites that are common to all the *Burkholderia* genomes. Homologous recombination is the *recA* driven housekeeping DNA repair process that replaces damaged DNA by using an undamaged sister strand as a template. This homologous recombination process can also drive evolution by causing the exchange of homologous gene regions between closely related cells and can result in both genotypic and phenotypic changes in the recipient. By definition, this process will preserve the original gene order except in cases in which illegitimate recombination introduces novel sequence along with the homologous regions [12]. Pearson et al. [8] found high recombination/mutation (r/m) ratios at MLST loci in *B*. *pseudomallei* e.g., r/m values ~ 25 in *B*. *pseudomallei* versus low r/m values of 2.91 in *Bacillis cereus* and 0.1 in *Staphlococcus aureus* [36]. These results suggest a significant level of homologous exchange within the *B*. *pseudomallei* genomes. Pearson et al. [8] calculated that values of the index of association are close to zero, suggesting that recombination in the species’ history has been common or that the species is not phylogenetically diverse [37]. We minimized the effect of the latter by using a geographically diverse panel of isolates.

The presence of two different kinds of genome altering activity (genomic island inserts and homologous recombination) might suggest that comparisons within the pan-genome might reflect a disorganized and complex system. But gene order estimations in this pan-genome study indicate the opposite, that the *B*. *pseudomallei* genomes do contain high degrees of co-linear relationships in gene order. It is worth reiterating that homologous recombination, as a repair function, maintains the integrity of the genomes by splicing an exact homolog into its original position and direction. It should, therefore, not be surprising that high levels of homologous recombination in the *B*. *pseudomallei* genome do not necessarily disrupt gene order within large stretches of its genome. Maintenance of gene order, which is generally conserved in bacteria only at very close phylogenetic distances [25], may be useful in maintaining the integrity of operons [38], but the scale at which gene order is conserved extends beyond single operons [39] even though gene order is not necessary for the functioning of groups of genes [40]. Thus, the high degree of gene order conservation seen in *B*. *pseudomallei* suggests that there may be selective pressures reducing recombination that alters the gene order.

Conversely, *Burkholderia pseudomallei*, characterized by a large, diverse collection of genomic islands driven by horizontal gene transfer, is also suited to large internal genomic rearrangements. This is a feature that could severely alter key spatial arrangements within the genome. The numerous tRNA, rRNA, and transposable sites within these genomes that are transferring and shuffling foreign DNA are also able to cause internal inversions at these same sites [6, 16]. Some of these inversions are already known to be 0.9 to 1.6 Mb in size [16]. What is not clear in any of these analyses is whether the extensive rearrangements in the *Burkholderia* genome are altering features in the genome such as the spatial relationship between the origin and termination site [41]. Several relatively recent studies suggest that there is a fundamental need to preserve the relative symmetry and orientation of the *ori* and *ter* sites in bacteria in order to maintain appropriate gene dosage [42–44]. In addition to a dosage gradient between the *ori* and *ter* sites, there is also a dosage effect between the 2 chromosomes [45] Due to the shorter replication time of the smaller chromosome, delayed initiation of replication ensures that termination is synchronous between the chromosomes [45]. This delayed replication increases the dosage of genes near the origin on the larger chromosome relative to the smaller, inhibiting interchromosomal rearrangements which would alter the relative dosage of particular genes [45]. Finally, due to their reduced expression, genes located on the smaller chromosome and further from the origins of replication tend to be under weaker purifying selection than those genes that are more highly expressed [46, 47]. Indeed, previous studies of *Burkholderia* have shown higher degrees of orthology and smaller ratios of nonsynonymous to synonymous substitutions on the larger chromosome and closer to the *ori* site [48]. The potential for extensive rearrangement of the *B*. *pseudomallei* genome provides an ideal setting to further examine the role of symmetry of the *ori* and *ter* sites or other rearrangements in the evolutionary history of the species.

## Conclusions

We have shown that the genome of *B*. *pseudomallei* is open, with each additional sequence beyond the 37 analyzed here expected to yield approximately 136 new genes to the pangenome. On average, 74% of the genes in a *B*. *pseudomallei* genome can be considered to be part of the extended core. This is lower than the 86% estimated by Sim et al. [7], a difference likely due to the geographically diverse (and therefore likely genetically diverse) strains we selected compared to those selected by Sim et al. [7]. By selecting diverse strains, we tried to encapsulate as much of the global pangenome as possible and therefore minimize additional diversity that may be found from additional strains. Conversely, the number of core genes is not expected to decrease much as more sequences are added, indicating that few genomes are likely to be found with ‘missing’ genes.

The core genome size itself is highly dependent on the definition of a core gene. We prefer a definition in the spirit of Lapierre and Gogarten [26], who suggested that the extended core be defined as genes present in at least 99% of strains. We argue, however, that even this is overly restrictive as over 100 strains would have to be sequenced to exclude even one, and such a definition fails to accommodate gene loss that significantly affects the biology of the organism, as it is thought to do in several strains of *B*. *pseudomallei* where gene loss is thought to preclude new human infection. By considering genes present in ≥35 of 37 genomes to be core genes, the estimated core genome size increases by almost 40% from 2,694 to 4,402 (Fig 4). Thus, this number includes genes from strains representative of the broad ecological niche occupied by *B*. *pseudomallei* but is not limited by the two strains restricted to highly specialized life styles.

Integration of DNA sequence into the *B*. *pseudomallei* genome is largely mediated by site-specific recombination at tRNA repeats and is often found at the edge of genomic islands [6]. However, the rest of the genome also shows evidence of extensive recombination [7, 8]. Because genomic islands typically contain many repeated sequences (particularly of phage genes), which make sequence assembly difficult, we could not determine directly whether the strain-specific or character genes were strictly present in genomic islands or distributed throughout the genome. The poor fit with models A and C described by Haegeman and Weitz [10] suggest that genes are not integrated into all regions of the genome equally (model A) nor is there a large region of the genome that is completely resistant to recombination (model C). The best fit to our data was provided by model D in which 96% of the genome undergoes very limited gene transfer and the remaining 4% of the genome undergoes extensive recombination.

Together, our findings that gene order is highly conserved in *B*. *pseudomallei* support a model in which a limited portion of the genome undergoes extensive gene transfer while the rest of the genome is relatively resistant. Conservation of gene order is incompatible with frequent horizontal gene transfer and recombination that disrupts gene order occurring anywhere in the genome. Rather, it is compatible with such events happening (perhaps repeatedly) at specific locations (such as GIs) or occurring in a reciprocal fashion. It is likely that both are occurring: high gene transfer at GIs is responsible for the large number of strain-specific and core genes observed here (and therefore the good fit with model D of Haegeman and Weitz, 2012), and homologous recombination of genes may account for the high levels of horizontal gene transfer observed at MLST sites [8].

Rare events that significantly rearrange the genome do occur, as shown by the large inversions present in some genomes [6, 16], however such inversions retain much of the original gene order as considered here because we measured gene order as the proportion of genes adjacent in one genome that are also adjacent in another. Thus, single large inversions only disrupt gene order at the chromosomal break points.

The frequency of rearrangements in bacteria may be limited by the availability of suitable insertion sequences, the rate of recombination, or the relative fitness of the rearranged genome [49]. The rate of recombination is largely driven by sequence similarity, with similar sequences recombining more frequently than diverse sequences, though the mechanism for controlling recombination varies among taxa [14, 15]. As a result, lateral gene transfer occurs primarily among closely related taxa [50, 51], though certain distant taxa may have notably high recombination rates [51]. The rate at which recombination and horizontal gene transfer occurs may be important in species formation and isolation [13, 52, 53].

The high frequency of recombination, as demonstrated by the diversity among strains, coupled with very high gene order conservation, suggests that such constraints may have been important in the genomic evolution of *B*. *pseudomallei*. High recombination rates have been observed in this species [8], and many potential insertion sequences (particularly associated with tRNAs) remain unexploited by GIs, leaving the unexplored possibility that rearrangements in the genome may limit individual fitness.

## Supporting Information

S1 File Accession numbers of sequences. (XLSX)

S2 File Proteins identified, their homology groups, and function. Proteins were identified by Prodigal, homology groups by OrthoMCL, and functions by myRAST.
(ZIP)
